# A single intravenous reelin injection restores corticosterone-induced neurochemical and behavioral alterations in dams during the post-partum period

**DOI:** 10.3389/fnmol.2024.1442332

**Published:** 2024-08-20

**Authors:** Carla L. Sánchez-Lafuente, Jenessa N. Johnston, Brady S. Reive, Kaylene K. A. Scheil, Ciara S. Halvorson, Mariana Jimenez, Darian Colpitts, Lisa E. Kalynchuk, Hector J. Caruncho

**Affiliations:** Division of Medical Sciences, University of Victoria, Victoria, BC, Canada

**Keywords:** reelin, post-partum depression, corticosterone, chronic stress, oxytocin, hippocampus, hypothalamus, neurogenesis

## Abstract

**Introduction:**

Treatment with the synaptic plasticity protein reelin has rapid antidepressant-like effects in adult corticosterone (CORT)-induced depressed rats, whether administered repeatedly or acutely. However, these effects remain unexplored in the context of post-partum depression (PPD).

**Methods:**

This study investigated the antidepressant-like effect of a single injection of reelin in a CORT-induced model of PPD. Long-Evans female dams received either daily subcutaneous CORT (40 mg/kg) or saline injections (controls) from the post-partum day (PD) 2 to 22, and on PD22 were treated with a single intravenous reelin (3 μg) or vehicle injection.

**Results:**

Reelin treatment fully normalized to control levels the CORT-induced increase in Forced Swim Test (FST) immobility and the decrease in reelin-positive cells in the subgranular zone of the intermediate hippocampus. It also increased the number of oxytocin-positive cells in the paraventricular nucleus (PVN), the number of reelin-positive cells in the dorsal and ventral hippocampus, and the dendritic complexity of newborn neurons in the intermediate hippocampus, causing a partial recovery compared to controls. None of these changes were associated with fluctuations in estrogen levels measured peripherally.

**Discussion:**

This study brings new insights into the putative antidepressant-like effect of peripherally administered reelin in an animal model of PPD. Future studies should be conducted to investigate these effects on a dose–response paradigm and to further elucidate the mechanisms underlying the antidepressant-like effects of reelin.

## 1 Introduction

Post-partum depression (PPD) is marked by the emergence of a depressive episode occurring within the first month up to a year after childbirth (DSM-5, 2022; Kroska and Stowe, 2020). While it is a common psychiatric condition afflicting up to 17% of mothers annually (Shorey et al., 2018) and causing profound, enduring repercussions on the welfare of their offspring (Takács et al., 2020), our grasp of the fundamental neurological mechanisms driving this disorder remains incomplete.

Current treatment approaches for PPD are largely aligned with those used for non-post-partum depression. Selective serotonin reuptake inhibitors are typically the initial choice. However, these medications have a protracted onset of therapeutic action, which can lead to suboptimal treatment outcomes and an elevated risk of adverse effects on the infant (De Crescenzo et al., 2014). More recent research has illuminated the potentially beneficial role of neurosteroids like allopregnanolone (Majewska et al., 1986; Almeida et al., 2020). However, allopregnanolone treatment in humans, necessitates an extended infusion period and controlled administration within a hospital setting, underscoring the urgent need for faster-acting therapeutics for this condition (Kanes S. J. et al., 2017; Kanes S. et al., 2017; Meltzer-Brody et al., 2018).

Reelin is an essential extracellular matrix protein, with significant roles in both brain development and synaptic plasticity in adulthood (Fatemi, 2005; Faini et al., 2021). Our previous research has indicated that both repeated and singular peripheral administrations of reelin yield fast-acting and prolonged antidepressant-like effects, ameliorating behavioral and neurochemical abnormalities caused by repeated CORT exposure (Allen et al., 2022; Scheil et al., 2024), similar to the effects observed with ketamine (Brachman et al., 2016; Johnston et al., 2020). Furthermore, there is evidence suggesting that reelin may modulate allopregnanolone levels in the rodent brain (Pinna et al., 2003). Based on these findings, we have formulated the hypothesis that reelin may also exhibit therapeutic potential preclinically by addressing depressive-like behaviors during the post-partum period.

In rat models, chronic administration of CORT for 3 weeks, results in a well-established depressive-like phenotype that parallels with the downregulation of reelin (Lussier et al., 2009; Sterner and Kalynchuk, 2010). Moreover, our investigations with this stressing paradigm have unveiled alterations in the density of reelin-positive cells in two crucial regions: the subgranular zone (SGZ) of the hippocampal dentate gyrus (DG) and the hypothalamic paraventricular nucleus (PVN) (Lussier et al., 2009, 2013; Brymer et al., 2018; Allen et al., 2022; Sánchez-Lafuente et al., 2022). A deficiency in reelin levels has been shown to increase susceptibility to the impact of chronic CORT exposure on despair-like behavior measured with the forced swim test (FST), as evidenced by studies involving heterozygous reeler mice (Lussier et al., 2011). During the post-partum period, estrogen levels decrease significantly and might contribute to the development of PPD (Hedges et al., 2021) and while estrogen can influence the reelin system and vice versa (Bender et al., 2010; Meseke et al., 2018) whether alterations in reelin occur during the critical post-partum phase and could be a vulnerability factor remains unexplored.

Disruptions in both the hypothalamic–pituitary–adrenal (HPA) axis and the oxytocin (OXT) system occur in PPD (Cyranowski et al., 2008). Patients often exhibit an irregular stress response and elevated cortisol levels (Taylor et al., 2009). Under normal conditions, OXT is released within the hypothalamus and the pituitary gland to establish an inhibitory influence on the HPA axis (Pati et al., 2019). However, when it comes to the impact of high CORT levels especially during the post-partum period on the stress-regulatory oxytocinergic activity is not fully clear. Recently, our group has found that a portion of parvocellular OXT-positive neurons within the PVN co-localize with reelin-positive cells. This co-localization pattern displays sex-specific characteristics and appears to be partially diminished following chronic CORT administration (Sánchez-Lafuente et al., 2022). This led us to hypothesize a potential link between reelin and the oxytocinergic system, which could play a crucial role in the pathophysiology of post-partum depression.

Building on this premise, our study was driven by two primary objectives. Firstly, to explore if depressive behavior in a corticosterone-induced model of post-partum depression aligned with reduced reelin in crucial brain regions regulating the HPA axis, such as the hypothalamic PVN and the hippocampal SGZ. Secondly, it sought to determine if a single peripheral injection of reelin could effectively mitigate the post-partum depression-like symptoms and neurochemical alterations induced by CORT. These findings offer initial insights into the potential role of reelin in both the development and treatment of post-partum depression.

## 2 Methodology

### 2.1 Animal husbandry and breeding

We used Long-Evans rats purchased from Charles River (QC, Canada). We purchased a total of 14 males, non-experienced breeders, and 28 females, primiparous. Rats were individually housed in rectangular polypropylene cages containing standard laboratory bedding with access to food and water *ad libitum* during a week of habituation and another week of handling. The rodent colony room was maintained at an ambient temperature of 20 ± 1°C on a 12:12 light–dark cycle (lights on at 7 am). Breeding was conducted as previously described (Workman et al., 2013). The experimental procedure was conducted following the guidelines of the Canadian Council on Animal Care, the National Institutes of Health guide for the care and use of laboratory animals and the University of Victoria Committee on Animal Care.

### 2.2 Experimental design

The post-partum depression animal model used in this study was based on the previously characterized model by Dr. Liisa Galea’s group (Workman et al., 2013). All dams were singly housed in a separate room and remained undisturbed until parturition except for cage changes and weighing. Day of birth was considered post-partum day (PD) 0. On PD 2, dams started receiving daily subcutaneous injections for 21 consecutive days. Rats in the CORT-vehicle (C/V, *n* = 7) and CORT-reelin (C/R, *n* = 7) received 40 mg/kg corticosterone 21-acetate (Sigma-Aldrich Canada, Oakville, Ontario), suspended in physiological saline with several drops of Tween 80 (Sigma-Aldrich). This dose was chosen based on previous publications (Gregus et al., 2005; Lussier et al., 2009). Rats in the saline-vehicle (S/V, *n* = 7) and saline-reelin (S/R, *n* = 7) received physiological saline (0.9% NaCl) with a few drops of Tween 80. All CORT and saline injections were based on body weight (1 mL/kg) and during the light phase of the light–dark cycle.

### 2.3 Reelin treatment

Recombinant reelin (R&D systems, 3,820-MR-025; composed of reelin repeats 3–6 and having a predicted molecular weight of 180 kDa by SDS PAGE using reducing conditions) was suspended in 0.5 mL of 0.1 M PBS (pH = 7.4) and administered intravenously on the lateral tail vein after the last CORT or saline injections (PD22). Rats in the S/R or C/R groups were treated with one dose of 3 μg/0.5 mL intravenous reelin and S/V and C/V with one dose of 0.5 mL of the same vehicle solution used for reelin injections. This was done by restraining rats with a DecapiCone (Braintree Scientific Inc., MA). Anesthesia was not used for this procedure. The specific dose of reelin was chosen based on its efficacy in restoring depressive-like phenotype in a similar animal model (Allen et al., 2022).

### 2.4 Estrous cycle

After each behavioral test, the estrous cycle stage was determined for the female rats by means of vaginal cytology samples as previously characterized (Long and Evans, 1922). Vaginal cytology samples were acquired by vaginal lavage with a pipette containing saline (0.9% NaCl). The contents of the pipette were then smeared onto a slide. The slides were dipped into 70% ethanol, allowed to dry, and later stained with Giemsa (Sigma-Aldrich). Each slide was examined under a light microscope, and the estrous cycle stage was determined by the presence of nucleated epithelial cells, cornified epithelial cells, or leukocytes.

### 2.5 Maternal behavior assessment

Maternal behavior was assessed following a previously published protocol (Champagne et al., 2001; Capone et al., 2005). Maternal observations were performed three times a day, at 10 am (light phase), 1 pm and 7 pm (dark phase). During each observation period, different behaviors of each mother were scored every 3 min for a total duration of 75 min (25observations per timepoint), with a total of 75 observations/day. Each behavior was scored as a discrete value (e.g., highest score per behavior is 75) and then transformed into a percentage. The scored behaviors can be classified as non-maternal behavior (off nest time, sleeping and self-grooming) and maternal behavior (active and passive nursing, licking and grooming the pups).

### 2.6 Behavioral testing

The Forced Swim Test was conducted on day 22 of the experiment during the light phase of the light–dark cycle using a one-day protocol (Marks et al., 2009). Each rat was placed in a rectangular Plexiglas tank with the dimensions of 25 cm × 25 cm × 60 cm for 10 min. The parameters determined were the time spent immobile and the latency to immobility.

The Elevated Plus Maze test was conducted on day 23, during the light phase of the light dark cycle. The apparatus (Noldus) used was 50 cm long × 10 cm wide × 75 cm tall. Rats were placed at the center junction of the maze facing an open arm and let them explore the maze for 5 min. Parameters measured include the number of entries into each arm and center and the distance traveled, performed by a researcher blind to treatment conditions. The distance traveled was automatically calculated with EthoVision^®^ XT 11.5 software (Noldus, Netherlands), but the rest was manually scored by someone blind to the experimental conditions. This protocol is based on the originally described by Pellow et al. (1985).

### 2.7 Perfusions and tissue preparation

On day 22 of the experiment, the rats were sacrificed with isoflurane and decapitation. Rats were perfused transcardially with 0.1 M phosphate buffer (PB, pH 7.4) followed by 4% (w/v) paraformaldehyde (PFA) in 0.1 M PB (pH 7.4) for 30 min. Brains were collected and kept on 4% (w/v) PFA in 0.1 M PB (pH 7.4) for 24 h at 4°C and then cryopreserved in 30% (w/v) sucrose in 0.1 M PB (pH = 7.4) for 48 h at 4°C until flash frozen with liquid nitrogen. Frozen brains were sectioned using a cryostat (Leica CM1850 UV), placed directly on slides for the PVN sections (20 μm) or on well plates with cryoprotectant for the hippocampus sections (30 μm), and stored at −20°C. Sections were distributed into series, separated by 6 sections, which was done following the Rat brain atlas coordinates (Paxinos and Watson, 2004); PVN sections (bregma −1.56 to −1.92 mm), SGZ dorsal (bregma −13.14 to −4.16), intermediate (bregma −4.16 to −5.2) and ventral (bregma 5.2 to 6.24) hippocampus (dHP, iHP, and vHP, respectively).

### 2.8 Immunolabeling

Brain sections in free-floating were rinsed for 5 min 3 times in 0.1 M Tris-buffered saline (TBS, pH 7.4), this was done between each of the following steps. First, we did antigen retrieval in sodium citrate (pH 6) at 85°C for 30 min. Then, incubated with the following primary antibodies: mouse anti-reelin (1:500, MILLIPORE, MAB5364) for 48 h at room temperature (RT), rabbit anti-DCX (1:1000, Cell Signaling Technologies, AB-561007) for 24 h at RT, and rabbit anti-oxytocin (1:1000, MILLIPORE, AB911) for 24 h at RT. All in a blocking solution containing 15% (v/v) normal goat serum (NGS), 0.5% Triton X-100, TBS with 1% Bovine Serum Albumin (BSA). The next step was to block endogenous peroxide activity with 10% (v/v) H_2_O_2_ for 30 min. Then, tissue was incubated at RT for 2 h with a biotinylated goat anti-mouse (GAM) secondary Ab (1:100 Vector Laboratories, United States, BA2001) diluted in a blocking solution. Then, tissue was incubated in avidin-biotin complex (ABC, 1:500, Vector Laboratories, United States) for 2 h at RT. For visualization, tissue was incubated with 0.05% (w/v) 30-diaminobenzidine (DAB, Sigma Aldrich, St. Louis, MO, PK6100), 0.0156% H_2_O_2_ diluted in TBS for 10 min, except DCX samples that were visualized with 4.167% NiSO4 and 0.002% H_2_O_2_. Finally, dehydration with ethanol gradient and xylenes was done before cover-slipping with Permount (Fisher Scientific SP15-500).

### 2.9 Cell counts

An unbiased method of counting called stereology was used to determine the number of reelin-positive cells immunolabeled with DAB by using a Nikon Eclipse E800 microscope with a motorized stage linked to a computerized image analysis program (Stereo Investigator, version 8.0, MicroBrightField Inc.). A total of 4 sections for the PVN (20 μm thick) and 5 sections for the SGZ (30 μm thick) were quantified including both hemispheres at 400× magnification. The total number of cells was calculated with the formula: *N*_total_: ΣQ^−^ × 1/ssf × A(x,y step)/a(frame) × t/h, where ΣQ^−^ is the number of counted cells; ssf is the section sampling fraction (1/6); A(x,y step) is the area associated with each x,y movement (10,000 μm^2^); a(frame) is the area of the counting frame (3,600 μm^2^); *t* is the weighted average section thickness; and h is the height of the dissector (9 μm for 20 μm tissue or 12 μm for 30 μm tissue) with a guard zone of 2 μm. Then *N*_total_ was divided by the total average area (traced while counting) resulting in a density value, this was done only for the PVN.

The DCX categorization was performed using a meander scanning approach, 100 randomly chosen DCX-IR cells were categorized into three groups based on dendritic complexity. Proliferative (no process or short process), intermediate (medium process with no branching), or post-mitotic (strong dendrite branching with multiple processes) based on previously published literature (Gobinath et al., 2016; Johnston et al., 2020).

### 2.10 Estradiol quantification

Plasma was isolated from samples containing 1:9 EDTA/Blood (collected from the heart during perfusions) and analyzed using the commercially available Estradiol Assay kit (Bio-techne, KGE014) to detect estradiol (estrogen) levels from plasma samples.

### 2.11 Statistical analyses

Statistical analysis was carried out using Statistical Package for the Social Sciences (SPSS) version 20 (IBM, United States). Data were tested for assumptions of normality and homogeneity of variance. Extreme outliers (values greater than 3xIQR’s) were omitted before carrying out appropriate statistical analyses and “out” values or outliers (values greater than 1.5xIQR’s) were only omitted when they affected normality (Shapiro–Wilk test) or homogeneity of the sample (Levene’s test). Group differences were considered statistically significant at *p* < 0.05 and data was expressed as mean ± 95%CI. The statistical test used for immunohistochemical and behavioral analysis when assumptions comply was a two-way ANOVA with Tukey *Post hoc*. ANCOVA analysis was performed for the treatment effects on the density of OXT-positive cells, to account for covariate effects of litter size. For body weight change analysis, since sphericity could not be assumed (*p* < 0.05) with Muchly’s test of Sphericity for a two-way-ANOVA repeated measures, the Greenhouse–Geisser correction was employed. Effect sizes are reported on the results as partial eta-squared (η^2^_p_) [small (0.01), medium (0.06), and large (0.14)] for ANOVA analyses. In addition, we report the percentage of recovery by reelin treatment, as calculated by the following formula: Percentage recovered = 100 − (CORT/Reelin mean − Saline/Vehicle mean) ÷ (CORT/vehicle mean − Saline/Vehicle mean) × 100.

## 3 Results

### 3.1 Chronic corticosterone during the post-partum period causes alterations in maternal behavior and body weight

**Figure 1:**
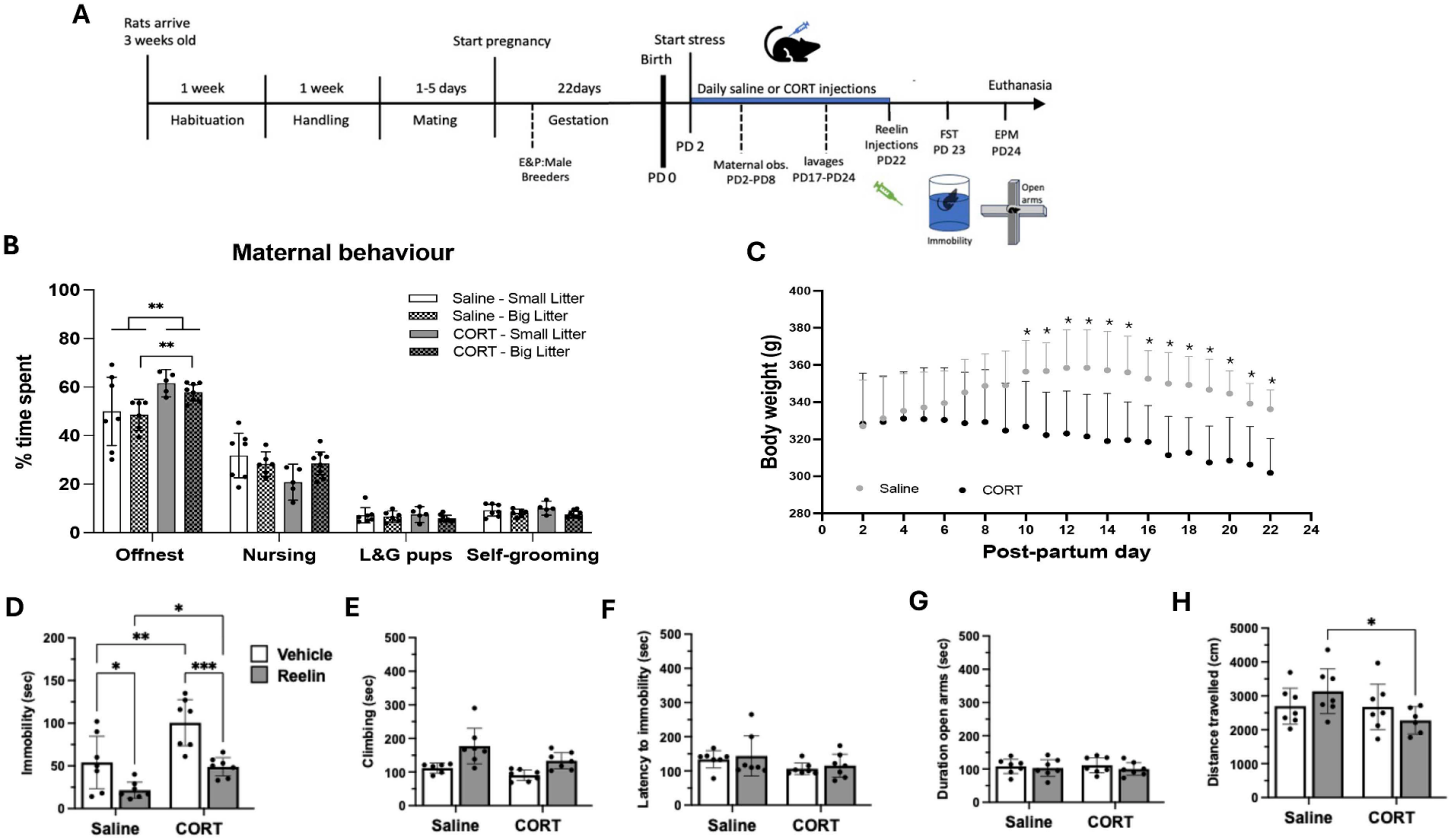
Body weight, maternal and post-partum behavior. **(A)** Experimental design. The chronic stress paradigm started on post-partum (PD) day 2. FST, forced swim test; EPM, elevated plus maze. **(B)** Average time spent on the different maternal behaviors between saline and CORT treated dams and dividing between big litter (>13pups) and small litter (=<13pups). Statistically significance **p < 0.01. **(C)** Change in body weight over time. Saline/Vehicle and Saline/Reelin groups are represented together into a single saline group and the same with CORT groups since reelin treatment occurred on the last day, hence not affecting these parameters. **(D–F)** Forced swim test behaviors: CORT increased immobility and was restored with reelin treatment. **(G,H)** Elevated plus maze behavior was not affected by either CORT or reelin treatment. Error bars on the graph represent Mean ± 95%CI and statistical significance *p < 0.05. *refers to the p value, with *p < 0.05, **p < 0.01, ***p < 0.001, ****p < 0.0001.

The CORT had significant effects on body weight, reducing weight gain over time compared to controls (Figure 1C). Repeated measures ANOVA analysis showed significant effects of time [*F*(1,20) = 11.203, *p* < 0.0001, η^2^_p_ = 0.483], effects of CORT [*F*(1,12) = 4.964, *p* = 0.046, η^2^_p_ = 0.293] and a combined effect of time and CORT [*F*(1,20) = 14.189, *p* < 0.0001, η^2^_p_ = 0.542] on the average body weight from PD9 to PD21, being significantly higher in saline than CORT-treated rats (*p* < 0.005) (Figure 1B).

Maternal behavior was assessed through observations performed during the first 8 days of CORT injections (Figure 1A). CORT treatment decreased maternal behavior, having a significant effect on the time spent off-nest [*F*(1,22) = 8.689, *p* = 0.007, η^2^_p_ = 0.283]. CORT-treated rats spent more time off-nest compared to saline rats (*p* = 0.0058) (Figure 1C). When looking at the effects of the litter size, we observe that it only affected the time spent self-grooming [*F*(1,22) = 5.020, *p* = 0.035, η^2^_p_ = 1.86]. Moreover, CORT-treated rats with a bigger litter (>=13 pups) spent more time offset (*p* = 0.004) compared to rats with a smaller litter (<13 pups) (*p* = 0.1323) (Figure 1C and Supplementary Table S1).

### 3.2 The corticosterone-induced alterations in FST behavior were restored with peripheral reelin while causing no changes in locomotor activity

Figures 1D–H shows the effects of repeated corticosterone on behavioral measures in the forced swim test and the elevated plus maze. Two-way ANOVA analyses showed effects of CORT on immobility [*F*(1,24) = 17.351, *p* = 0.0001, η^2^_p_ = 0.420] and climbing [*F*(1,24) = 7.108, *p* = 0014, η^2^_p_ = 0.249] in the FST but no effects on the latency to immobility or on swimming. Also, peripheral reelin treatment affected immobility [*F*(1,24) = 22.520, *p* = 0.0001, η^2^_p_ = 0.484] and climbing [*F*(1,24) = 7.95, *p* = 0.009, η^2^_p_ = 0.228] (Figures 1D–F and Supplementary Table S2). There was a significant increase in immobility in the FST in rats stressed with repeated CORT compared to saline/vehicle (*p* = 0.0019) and saline/reelin (*p* = 0.001). Moreover, rats treated with a single peripheral reelin injection spent the same time immobile as the Saline/Vehicle group and significantly less time than the CORT/Vehicle rats (*p* = 0.0007). No effects of either CORT or reelin on the time spent in the open arms or distance traveled in the EPM (Figures 1G,H and Supplementary Table S2).

**Figure 2:**
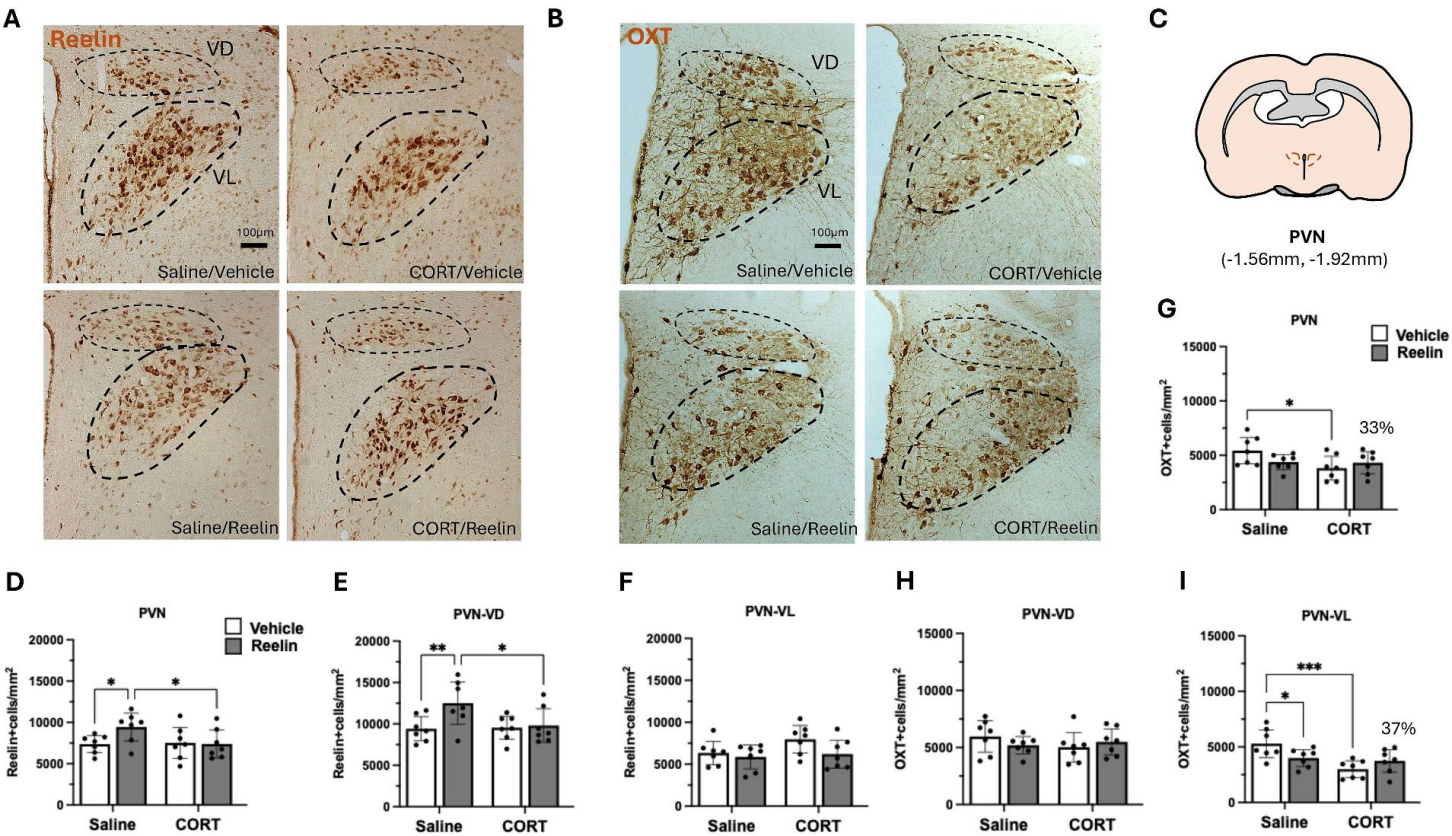
Oxytocin and reelin immunohistochemistry results in the PVN. **(A,B)** Representative immunostainings of reelin-positive cells **(A)** and oxytocin **(B)** in the PVN, lines mark ventrodorsal (VD) and ventrolateral (VL) subdivisions, from a coronal view. Scale bar is 100 μm. **(C)** Representation of the paraventricular nucleus of the hypothalamus in a coronal section view. **(D–F)** Average density of reelin-positive cells in the PVN, separated by VD and VL divisions. No changes in Reelin-positive cells. **(G–I)** Average density of oxytocin-positive cells in the PVN, separated by VD and VL divisions. Reelin treatment partially restored 33% of the oxytocin-positive cells in the PVN (37% in the VL division). Error bars show Mean ± 95%CI and statistical significance *p < 0.05. *refers to the p value, with *p < 0.05, **p < 0.01, ***p < 0.001, ****p < 0.0001.

### 3.3 Neither chronic CORT nor peripheral reelin injections caused significant changes in PVN reelin-positive neurons

**Figure 3:**
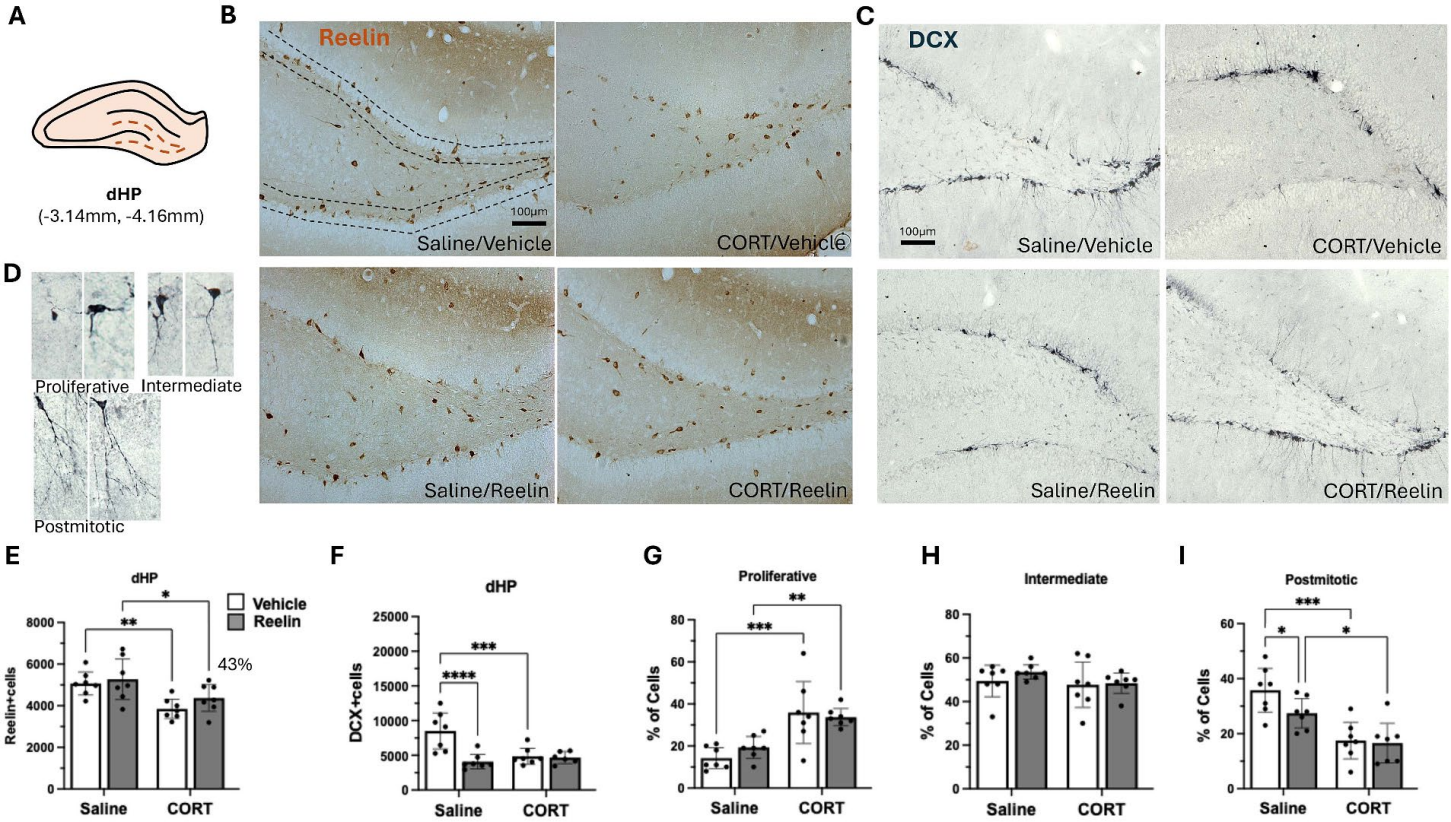
Dorsal hippocampal SGZ immunohistochemistry results. **(A)** Representation of dorsal hippocampus (dHP) section in a coronal view. **(B,C)** Representative images of immunostaining against reelin **(B)** and DCX **(C)** in coronal sections of the dHP. Dotted lines delineate the SGZ of the dentate gyrus. The scale bar is 100 μm. **(D)** Representative images of DCX-positive cells in the different maturation stages, categorized into proliferative, intermediate and postmitotic. **(E)** Average number of reelin-positive cells in dHP, was significantly decreased by CORT and partially recovered 43% with reelin treatment. **(F)** Average number of DCX-positive cells, and **(G–I)** the percentage of DCX-positive cells in proliferative **(G)**, intermediate **(H)** and postmitotic state **(I)**. Error bars show Mean ± 95% CI and statistical significance *p < 0.05. *refers to the p value, with *p < 0.05, **p < 0.01, ***p < 0.001, ****p < 0.0001.

Chronic CORT and subsequent treatment with peripheral reelin had no effects on the density of reelin-positive cells in the PVN (Figure 2). Two-way ANOVA showed a small effect of reelin treatment on the density of reelin-positive cells in the PVN ventrodorsal division [*F*(1,24) = 4.590, *p* = 0.043, η^2^_p_ = 0.101] but no effects of CORT. *Post hoc* multiple comparison analysis shows that saline/reelin rats had a significantly higher density of reelin-positive cells in the PVN-VD compared to saline/vehicle rats (*p* = 0.009), yet these group differences were not observed in the ventrolateral (VL) region (Figure 3F and Supplementary Table S3).

### 3.4 Corticosterone at post-partum caused a decrease in PVN oxytocin-positive cells that was partially restored with a single peripheral reelin injection

Due to the putative effects of the litter size on oxytocin, we performed a two-way ANCOVA analysis with litter size (small vs. big) as covariate. This showed that the litter size only had a covariate effect in the density of OXT-positive cells in the ventrodorsal division [*F*(1,24) = 5.897, *p* = 0.023, η^2^_p_ = 0.204] but not on the ventrolateral and thus not on the overall PVN population. Analysis of between-subject effects showed that both CORT [*F*(1,24) = 42.004, *p* = 0.0001, η^2^_p_ = 0.646] and reelin [*F*(1,24) = 11.668, *p* = 0.002, η^2^_p_ = 0.337] had a significant effect on the ventrolateral PVN, with no interaction effects. CORT/Vehicle rats had a lower density of oxytocin-positive cells compared to saline/vehicle (*p* = 0.0366). These effects disappeared when looking at both the total PVN with both the VD and VL populations. Moreover, as observed in Figure 2G, reelin treatment caused a 33% increase in the density of oxytocin-positive cells in the entire PVN and 37% in the VL division (Figure 2I), compared to CORT/vehicle. Since the CORT/Reelin group was not significantly different from either CORT/Vehicle (PVN, *p* = 0.689; PVN-VL, *p* = 0.0572) or saline/vehicle (PVN, *p* = 0.286; PVN-VL, *p* = 0.641), this was considered a partial recovery (Supplementary Table S4).

### 3.5 A single peripheral reelin injection partially restored CORT-induced decreases in reelin-positive cells in the dHP, fully restored them in the iHP and no changes in vHP

**Figure 4:**
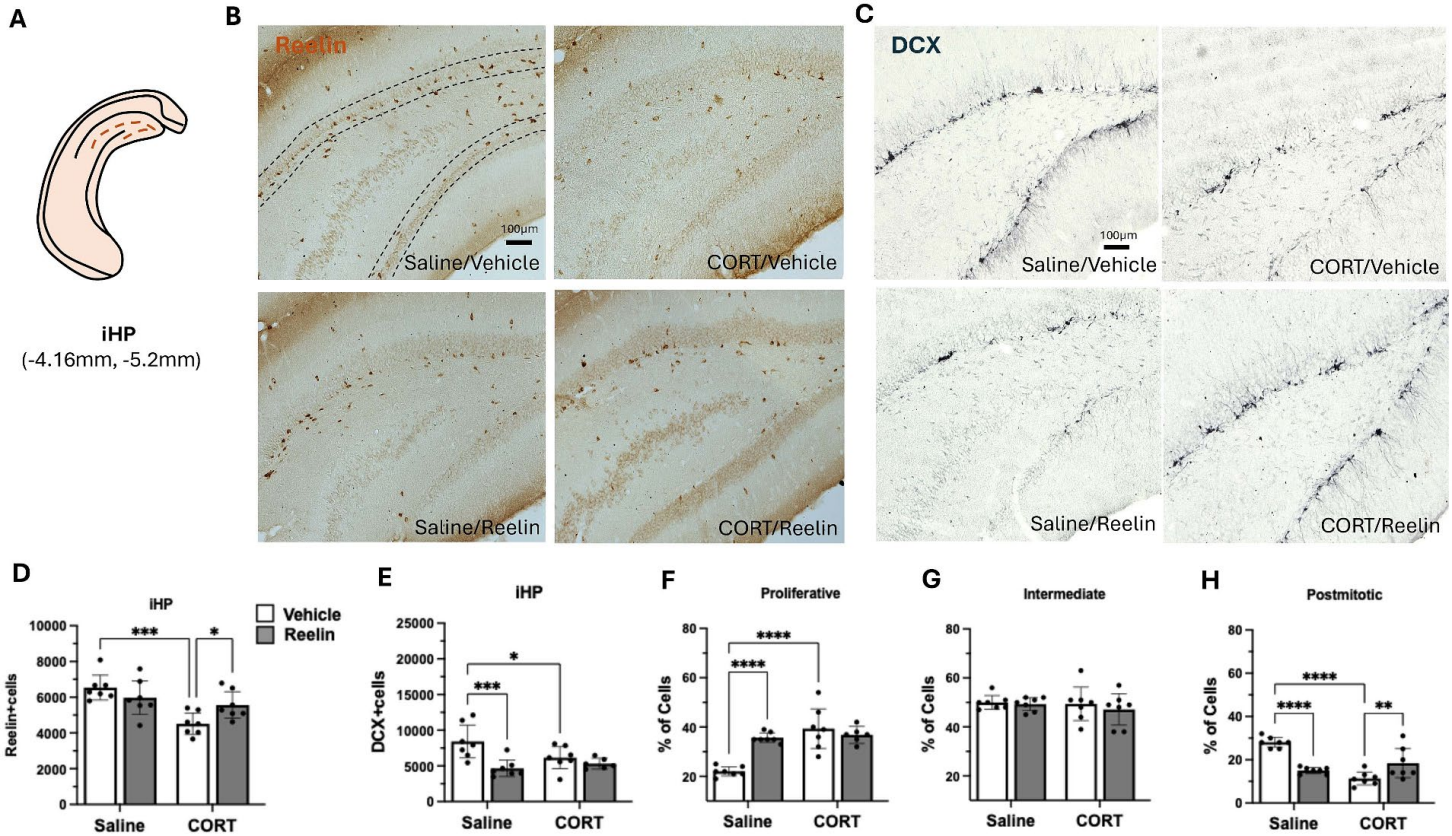
Intermediate hippocampal SGZ immunohistochemistry results. **(A)** Representation of intermediate hippocampus (iHP) section in a coronal view. **(B,C)** Representative images of immunostaining against reelin **(B)** and DCX **(C)** in coronal sections of the iHP. Dotted lines delineate the SGZ of the dentate gyrus. The scale bar is 100 μm. **(D)** Average number of reelin-positive cells, was significantly decreased by CORT and fully recovered with reelin treatment. **(E)** Average number of DCX-positive cells, and **(F–H)** the percentage of DCX-positive cells in proliferative **(F)**, intermediate **(G)**, and postmitotic state **(H)**. Error bars show Mean ± 95%CI and statistical significance *p < 0.05. *refers to the p value, with *p < 0.05, **p < 0.01, ***p < 0.001, ****p < 0.0001.

**Figure 5:**
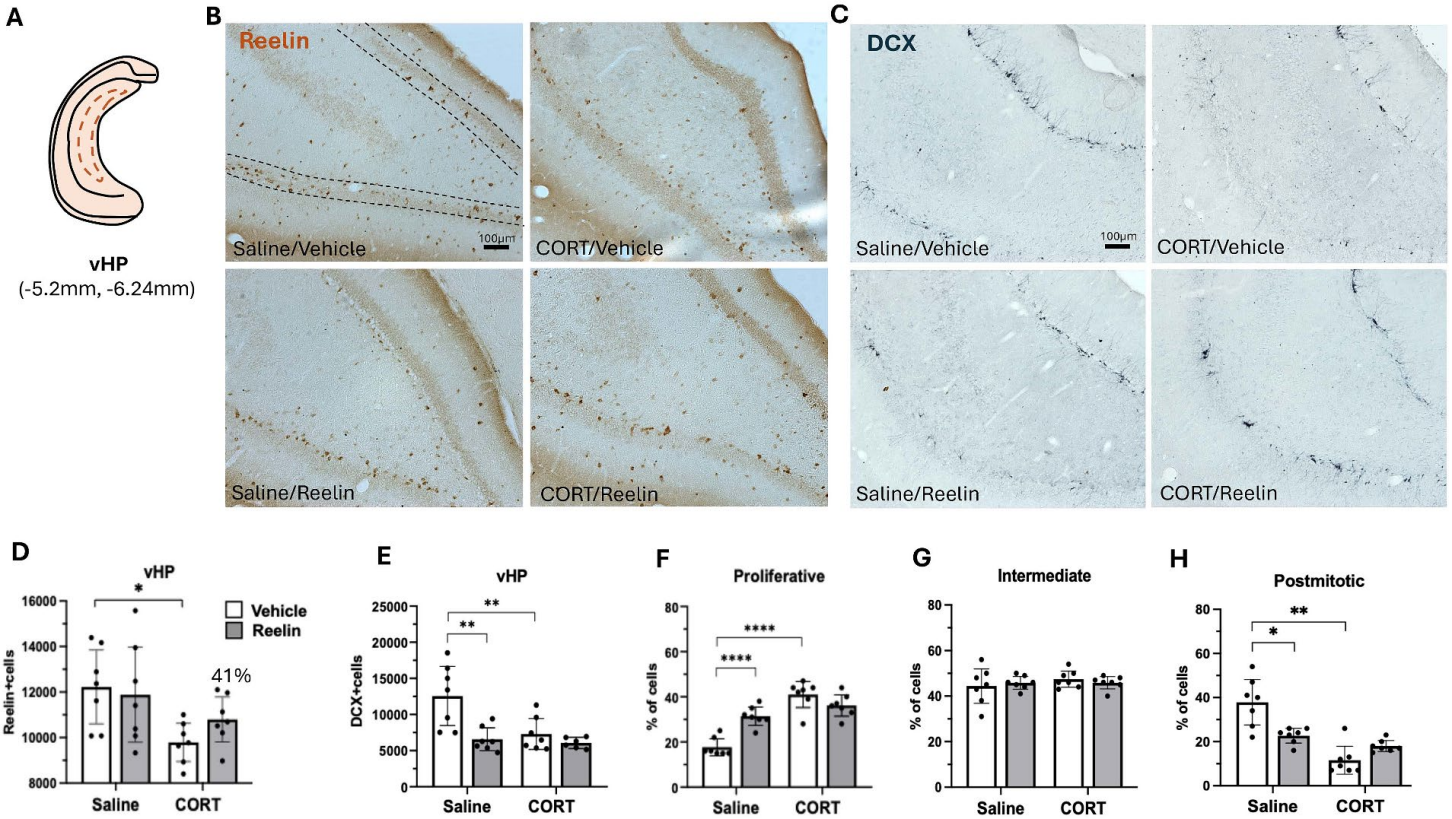
Ventral hippocampal SGZ immunohistochemistry results. **(A)** Representation of ventral hippocampus (vHP) section in a coronal view. **(B,C)** Representative images of immunostaining against reelin **(B)** and DCX **(C)** in coronal sections of the iHP. Dotted lines delineate the SGZ of the dentate gyrus. The scale bar is 100 μm. **(D)** Average number of reelin-positive cells, was significantly decreased by CORT and partially recovered 41% with reelin treatment. **(E)** Average number of DCX-positive cells, and **(F-H)** the percentage of DCX-positive cells in proliferative **(E)**, intermediate **(F)** and postmitotic state **(G)**. Error bars show Mean ± 95%CI and statistical significance *refers to the *p* value, with *p < 0.05, **p < 0.01, ***p < 0.001, ****p < 0.0001.

Two-way ANOVA analyses showed that CORT caused a significant effect on SGZ reelin-positive cells in the dHP [*F*(1,24) = 14.280 *p* = 0.001, η^2^_p_ = 0.373], and while peripheral reelin treatment did not have a significant effect, a partial restoration of 43% reelin-positive cells was observed. *Post hoc* analyses showed that CORT/Reelin animals were not significantly different from CORT/saline (*p* = 0.5575) nor Vehicle/Saline (*p* = 0.3190) (Figures 3B,D). Similarly, CORT affected iHP reelin-positive cells [*F*(1,24) = 15.592, *p* = 0.001, η^2^_p_ = 0.394] with an interaction effect with reelin [*F*(1,24) = 6.912, *p* = 0.015, η^2^_p_ = 0.224]. In this case, reelin treatment fully restored reelin-positive cells as CORT/Reelin was significantly different from CORT/Vehicle (*p* = 0.024) (Figures 4B,D and Supplementary Table S3). In the vHP, CORT had a significant effect on reelin levels [*F*(1,24) = 8.571, *p* = 0.007, η^2^_p_ = 0.263], but no effects of reelin alone or in combination with CORT. *Post hoc* analysis revealed that CORT/vehicle dams had significantly fewer reelin-positive cells in the vHP compared to saline/vehicle rats (*p* = 0.040) and this was partially restored (41%) with a reelin injection (*p* = 0.358) (Figures 5B,D and Supplementary Table S3).

### 3.6 A single peripheral reelin injection partially restored CORT-induced decreases in dendritic complexity only in the iHP

Changes in neurogenesis were analyzed with the number of DCX-positive cells and dendritic complexity was assessed by the categorization of neurons as described in the methods and observed in Figure 1D. CORT significantly decreased the number of DCX-positive cells in the dHP (*p* = 0.006) but it was not restored with reelin treatment (*p* = 0.8506), instead, reelin decreased the number of DCX-positive cells in saline rats (*p* = 0.002) compared to controls [*F*(1,24) = 6.023, *p* = 0.022, η^2^p = 0.201]. CORT also reduced dendritic complexity, by increasing the % of proliferative cells (*p* = 0.002) and decreasing the % of postmitotic cells (*p* = 0.0001). Reelin did not significantly improve dendritic complexity in the dHP (*p* > 0.05) (Figures 1C,F–H and Supplementary Table S5).

In the iHP, reelin [*F*(1,24) = 8.308, *p* = 0.008, *η*^2^_p_ = 0.257] and CORT in combination with reelin [*F*(1,24) = 6.538, *p* = 0.017, *η*^2^_p_ = 0.214] had an effect on DCX-positive cells. CORT decreased the number of DCX-positive cells but reelin treatment did not restore it. CORT significantly increased the % of proliferative cells compared to controls (*p* < 0.001). Reelin significantly increased the % of postmitotic cells (*p* = 0.021) compared to CORT/vehicle, yet still not fully recovered to match control levels (*p* = 0.002) (Figures 4C,E–G and Supplementary Table S6).

In the vHP, the number of DCX-positive cells was significantly decreased by CORT [*F*(1,23) = 7.565, *p* = 0.011, *η*^2^_p_ = 0.248], reelin [*F*(1,23) = 11.832, *p* = 0.002, *η*^2^_p_ = 0.340] and in combination CORT & Reelin [*F*(1,23) = 5,094, *p* = 0.034, *η*^2^_p_ = 0.181]. *Post hoc* showing that CORT/vehicle (*p* = 0.007), saline/reelin (*p* = 0.002) and CORT/reelin (*p* = 0.001) had significantly fewer DCX-positive cells than saline/vehicle. From these DCX-positive cells, the % of proliferative cells was significantly affected by CORT [*F*(1,24) = 54.698, *p* < 0.001, *η*^2^_p_ = 0.695], reelin [*F*(1,24) = 5.473, *p* = 0.28, *η*^2^_p_ = 0.186] and both [*F*(1,24) = 24.063, *p* < 0.001, *η*^2^_p_ = 0.501]. *Post hoc* showed that saline/vehicle dams had significantly fewer proliferative cells compared to the other groups (*p* < 0.001). Similarly, when looking at the % of postmitotic cells, there was a significant effect of CORT [*F*(1,24) = 35.281, *p* < 0.0001, *η*^2^_p_ = 0.595], and an interaction effect of reelin with CORT [*F*(1,24) = 2.788, *p* < 0.001, *η*^2^_p_ = 0.416]. *Post hoc* showed that saline/vehicle dams had significantly more postmitotic cells compared to the other groups (*p* < 0.01). No effects on the % of intermediate cells (Figures 5C, E–G and Supplementary Table S7).

### 3.7 Effect of the estrous cycle and peripheral estrogen levels on behavioral and immunohistochemical measures

The stage of the estrous cycle was determined from vaginal swabs performed after FST (PD23) and EPM (PD24). Then we looked at the distribution of estradiol levels among the estrous cycle between different treatment groups (Supplementary Figure S1B) but the distribution of animals among the different stages of the estrus cycle was too small to extract any general correlations. Moreover, estrogen (estradiol) levels did not significantly differ between CORT or saline treatment groups (Supplementary Figure S1A).

### 3.8 Correlations between immunohistochemical and behavioral measures

Oxytocin levels in the PVN-VL did not correlate with immobility in the FST but they did positively correlate with the time spent off-nest (decreased maternal behavior) in saline-treated rats (*R* = 0.759, *p* = 0.0026). The density of oxytocin-positive cells in the PVN positively correlated with the density of reelin-positive cells in the saline/reelin group (*R* = 0.832, *p* = 0.020) so the more oxytocin the more reelin (Table 1). The number of reelin-positive cells in the iHP positively correlated with the number of DCX-positive cells in saline/vehicle (*R* = 0.800, *p* = 0.031) and saline/reelin rats (*R* = 0.801, *p* = 0.030), so the more reelin the more DCX-positive cells (Table 2).

**Table 1:** Correlations with the density of oxytocin-positive cells in the PVN-VL.

Comparison	Group	*n*	Pearson’s *R*	*p-*value
FST immobility	Saline/vehicle	7	−0.2918	0.5255
	CORT/vehicle	7	−0.2086	0.6535
	Saline/Reelin	7	0.6554	0.1100
	CORT/Reelin	7	0.006433	0.9891
Non-maternal behavior (off-nest)	Saline	14	0.7594	0.0026**
	CORT	14	−0.1254	0.6831
Reelin	Vehicle/vehicle	7	−0.0279	0.9527
	CORT/vehicle	7	−0.693	0.0841
	Vehicle/Reelin	7	0.832	0.0201*
	CORT/Reelin	7	0.521	0.2303

*Indicates a statistically significant value. *refers to the p value, with *p < 0.05, **p < 0.01, ***p < 0.001, ****p < 0.0001.

**Table 2:** Correlations with the density of reelin-positive cells in the iHP.

Comparison	Group	*n*	Pearson’s *R*	*p-*value
FST Immobility	Saline/vehicle	7	−0.1907	0.6821
	CORT/vehicle	7	−0.4623	0.2963
	Saline/reelin	7	−0.5469	0.2040
	CORT/reelin	7	−0.1483	0.7510
DCX-positive cells iHP	Saline/vehicle	7	0.8006	0.0305*
	CORT/vehicle	7	−0.3880	0.3898
	Saline/reelin	7	0.8016	0.0301*
	CORT/reelin	7	−0.1874	0.7222
% postmitotic cells iHP	Saline/vehicle	7	0.3040	0.5075
	CORT/vehicle	7	0.4105	0.3603
	Saline/reelin	7	−0.4491	0.3121
	CORT/reelin	7	−0.3885	0.3890

*Indicates a statistically significant value.

## 4 Discussion

This study shows that a single intravenous injection of reelin reverses some of the neurochemical and behavioral changes induced by corticosterone in post-partum dams. Using the previously established corticosterone-induced model of post-partum depression (Workman et al., 2013) we found that daily chronic corticosterone injections (40 mg/kg) to the dams from day 2 to day 24 of the post-partum period decreased the density of reelin-positive cells, DCX-positive and dendritic complexity in the dorsal and intermediate hippocampal SGZ (Figures 1, 4, 5). Moreover, decreased oxytocin-positive cells in the hypothalamic PVN (Figure 3), paralleling increases in despair-like behavior in the FST (Figure 2). Treatment with a single peripheral injection of reelin (3 μg dose), was able to fully restore CORT-induced behavioral alterations in the FST (Figure 2), hippocampal reelin levels mainly in the intermediate hippocampus (Figure 4) while partially restoring the density of oxytocin-positive cells in the PVN ventrolateral division (Figure 1) and neuronal maturation in the intermediate hippocampus (Figure 4).

### 4.1 Post-partum CORT treatment reduced body weight and maternal behavior

The reduced weight gain noted in dams treated with high levels of CORT during the post-partum phase aligns with findings from other studies involving post-partum or virgin rats (Johnson et al., 2006; Brummelte and Galea, 2010; Allen et al., 2022). In humans, weight loss is linked to depressive symptoms, and changes in weight and appetite are recognized as individual symptoms of depression (DSM-5, 2022). Regarding maternal behavior, we found that CORT-treated dams spent more time off-nest, which is a sign of decreased maternal behavior. Notably, though, dams with smaller litter sizes displayed a greater range of maternal behaviors, which, in turn, made the impacts of CORT less conspicuous when compared to dams with larger litter. This difference is likely attributable to variations in baseline stress levels arising from the care of more pups. Similar to our findings, other studies have shown that dams exposed to CORT during the post-partum period spend notably less time on the nest and nursing compared to control dams (Brummelte et al., 2006; Brummelte and Galea, 2010). Additionally, dams displaying more depressive-like behavior at the post-partum period engage in less active nursing compared to dams with lower levels of depressive-like behavior (Kurata et al., 2009).

### 4.2 Reelin treatment restored despair-like behavior in the FST but no changes in EPM

Consistent with existing literature (Brummelte and Galea, 2010; Gobinath et al., 2018), repeated CORT during the post-partum phase increased immobility in the forced swim test. The fact that we did not see any significant differences in the EPM suggests that the difference in the forced swim test is not due to general changes in locomotor activity in the CORT-treated animals. Noteworthy, a single injection of reelin completely reversed the immobility observed in the FST, which is a novel finding. Previous studies have indicated that fluoxetine alone was insufficient to alleviate despair-like behavior in the FST during the post-partum period; success was only achieved when combined with voluntary running (Workman et al., 2016; Gobinath et al., 2018). Moreover, while we did not see an increase in anxiety-like behavior produced by CORT, reelin also did not cause a change in this behavior, unlike the previous study where fluoxetine increased anxiety-like behavior in the open field test (paralleling side effects observed in humans). Thus, the ability of reelin alone to produce such antidepressant effects is promising, suggesting a putative use of reelin as a rapid-acting antidepressant comparable to ketamine to treat post-partum depression, with potentially better efficacy and safety profile than fluoxetine.

### 4.3 Reelin treatment partially restored oxytocin levels in the PVN

As previously observed by our group, chronic CORT treatment caused a decrease in OXT-positive neurons in the PVN (Sánchez-Lafuente et al., 2022), yet this time was observed during the post-partum in dams. A downregulation of oxytocin is also found in women showing depressed symptoms at post-partum and who have high CORT levels (Cox et al., 2015). Moreover, we observed a partial recovery of PVN oxytocin levels upon reelin treatment. Oxytocin alterations have been associated with the development of depression during the peripartum period (Skrundz et al., 2011; Stuebe et al., 2013) and previous research has associated the restoration of PVN oxytocin levels with the reversal of stress-induced depressive-like behaviors (Purba et al., 1996; Yamamoto et al., 2004; Meynen et al., 2007). In our study, OXT-positive cells in the PVN did not correlate with immobility behavior in the FST suggesting although the PVN may play a role in the development of despair-like behavior, other areas such as the hippocampus, also contribute to despair-like behavior following chronic stress. The extent to which PVN oxytocin mediates the antidepressant-like effect of peripheral reelin treatment remains uncertain following this study.

Additionally, while prior research has shown that estrogen can influence reelin and oxytocin levels (Shughrue et al., 2002; Hedges et al., 2021) and enhances HPA axis response to stress (Isgor et al., 2003; Figueiredo et al., 2007), alterations to estrogen did not significantly affect these parameters within our animal model. It is important to note that peripheral and central hormonal measures do not always align (Valstad et al., 2016), therefore further investigation of central nervous system (CNS) estrogen is warranted to comprehensively evaluate the role of estrogen in this model. Breastfeeding and nursing, which can depend on litter size, can also affect the oxytocinergic tone in the PVN (Ludwig and Leng, 2006). We observed that litter size affected OXT levels in the ventrodorsal division, potentially explaining why no effects of CORT or treatment were found in this region. The dorsal region contains more parvocellular neurons, whereas the lateral division contains more magnocellular neurons, each with distinct functions. This difference might explain the lack of litter size effects on OXT levels in the ventrolateral division. Nonetheless, litter size differences did not obscure the effects of CORT and reelin treatment on the ventrolateral OXT population.

### 4.4 Peripheral reelin treatment restored hippocampal reelin and partially restored dendritic complexity of immature neurons

While we have previously seen that chronic CORT treatment decreases the number of reelin-positive cells in the hippocampus SGZ in adult male and female rats (Brymer et al., 2020; Allen et al., 2022; Johnston et al., 2023; Reive et al., 2024), this was limited to the dorsal hippocampus and nulliparous virgin rats. Our group has also shown previously either intraventricular or intravenous injection of reelin restores reelin cell counts in the hippocampal SGZ following chronic CORT treatment (Brymer et al., 2020; Allen et al., 2022; Johnston et al., 2023; Reive et al., 2024). Here, we replicated both findings in PPD dams, showing that exogenous reelin restores CORT-induced changes in endogenous reelin levels.

We also found that CORT affected both neurogenesis (DCX-positive cells) and dendritic complexity of immature neurons in the hippocampal SGZ but more significantly in the intermediate rather than the dorsal region, which could be explained by the fact that newborn cells in the iHP are more sensitive to glucocorticoids (Levone et al., 2021) which regulate cell proliferation, survival, and differentiation. Differences in CORT effects between the dorsal and ventral hippocampus align with studies conducted by Galea’s group with the same animal model (Brummelte and Galea, 2010; Workman et al., 2016; Gobinath et al., 2018). Furthermore, reelin treatment partially restored dendritic complexity, leading to a shift in DCX-expressing cells toward a postmitotic stage of development. This outcome was anticipated given that overexpression of reelin is known to result in dendritic hypertrophy (Pujadas et al., 2010; Teixeira et al., 2011).

In prior studies using adult nulliparous rats, we observed that repeated peripheral reelin injections partially countered the CORT-induced decreases in DCX-positive cells and dendritic complexity within the SGZ. In the current study with post-partum dams, we observed similar effects with a full recovery in more matured neurons in the intermediate hippocampus, indicating that reelin’s influence was also significant during this period. Interestingly, other groups have seen that fluoxetine failed to restore DCX-positive cells in either the dorsal or ventral hippocampus (Workman et al., 2016; Gobinath et al., 2018) similarly observed here. Therefore, our findings highlight similar effects of reelin and fluoxetine in mitigating the impact of CORT on hippocampal neurogenesis.

### 4.5 Peripheral reelin as a putative antidepressant treatment for post-partum depression

Reelin is an extracellular matrix protein whose expression is epigenetically regulated and can be influenced by environmental factors (Chen et al., 2002). Both insufficient and excessive levels of reelin have been associated with pathological states (Teixeira et al., 2011; Alexander et al., 2023; Calvier et al., 2023), indicating that maintaining homeostatic levels of reelin may be critical for healthy functioning. Consequently, treatment with exogenous reelin is not straightforward and requires careful consideration of the therapeutic range to avoid adverse effects. For example, previous observations indicated that multiple reelin injections failed to restore CORT-induced reductions in spleen white pulp, whereas a single injection was fully effective (Reive et al., 2024). Additionally, an acute injection of 3 μg was found to be as effective or better than multiple injections in reversing behavioral and neurochemical alterations induced by CORT, underscoring the dose-dependent actions of reelin (Allen et al., 2022).

The hippocampus plays a crucial role in regulating HPA responsiveness to stress through inhibitory projections to the PVN (Herman et al., 2005). While we did not observe alterations in reelin levels at the PVN, changes in oxytocin were noted, possibly due to shifts in inhibitory tone from the hippocampus influenced by reelin levels. Additionally, peripheral oxytocin administration has been shown to promote neurogenesis in the hippocampus (Leuner et al., 2012), and reductions in oxytocin levels have been linked to symptoms of PPD suggesting a protective role of oxytocin in PPD (Mughal et al., 2024). However, modulations of the PVN oxytocinergic activity might also be partially involved in the underlying antidepressant actions of reelin treatment and other mechanisms of action like changes in hippocampal neuronal maturation could be involved.

We have shown previously the blockade of AMPA receptor activity with its antagonist CNQX ameliorates the antidepressant-like effects of reelin treatment and reelin restores long-term potentiation (LTP) in the hippocampus following chronic stress, suggesting regulation of synaptic plasticity is a key mechanism through which reelin administration reverses depression-like behavior (Brymer et al., 2020; Johnston et al., 2023). Supporting this possibility, ketamine which also shows therapeutic efficacy for PPD (Alipoor et al., 2021), has been shown to restore synaptic plasticity suggesting restoring synaptic plasticity is core to the recovery of depressive symptoms (Piazza et al., 2024). Ketamine has also been shown to restore central oxytocin levels, influence reelin expression in the hippocampal SGZ, and modify dendritic complexity (Meseke et al., 2018; Johnston et al., 2020; Zhu et al., 2021) and this study together with previous from our group have shown the same effects with reelin treatment (Johnston et al., 2023; Scheil et al., 2024), further supporting the predictive validity of the animal model and suggesting the potential antidepressant benefits of reelin for PPD treatment.

It is important to acknowledge that in this study, we administered the central fragment of recombinant reelin, which is not detected by the antibody used, as this antibody tags the N-terminal of reelin that is absent from the central fragment administered. This suggests the administration of recombinant reelin upregulated endogenous reelin levels in the hippocampus as observed in our immunohistochemistry evaluation (see Figures 1, 4, 5). Additionally, we did not visualize the administered reelin fragment to determine whether it crossed the blood–brain barrier (BBB) to act directly on nervous tissue. While this study contributes to the body of literature demonstrating that neurochemical changes in the CNS can be achieved through peripheral treatment with reelin (Allen et al., 2022; Jin et al., 2022; Johnston et al., 2023; Scheil et al., 2024), future studies need to determine whether these CNS changes occur indirectly through peripheral modifications or directly via crossing the BBB.

It remains unclear whether reelin can cross the BBB. While reelin has been identified in caveolar vesicles of brain endothelial cells, indicating the possibility that reelin or its fragments might be transported across the BBB (Perez-Costas et al., 2015), a more likely scenario is that reelin induces peripheral changes that affect the central nervous system (CNS). Peripheral reelin affects physiological processes by regulating inflammatory mediators and maintaining endothelial homeostasis. It decreases the expression of leukocyte-endothelial adhesion proteins, thereby reducing inflammatory cell recruitment and promoting endothelial homeostasis via ApoER2 receptors and NF-κB pathways (Ramesh et al., 2011; Ding et al., 2016). Studies on RELN+/− mice and CORT-treated rats indicate that reelin treatment can partially or fully restore SERT cluster abnormalities in blood lymphocytes (Rivera-Baltanas et al., 2010, 2012, 2015; Romay-Tallon et al., 2018; Caruncho et al., 2019) and reverse spleen white pulp atrophy in CORT-treated rats (Reive et al., 2023, 2024). These studies show that reelin in the periphery alleviates lymphocyte dysfunction and specific depression-related inflammatory phenotypes. Therefore, we could speculate that peripheral reelin treatment might modulate the peripheral immune system. Peripheral immune changes can translate to the CNS (Fani Maleki and Rivest, 2019; Greenhalgh et al., 2020) and could potentially lead to alterations in gene expression, synaptic plasticity, neurogenesis, among other neurochemical changes, ultimately influencing brain neurochemistry involved in behaviors such as despair-like behavior (Baitsch et al., 2011; Brymer et al., 2018). It is however of interest to point out that in our conventional repeated-CORT paradigm in adult rats one single peripheral injection of recombinant at reelin can reverse several behavioral and neurochemical changes induced by repeated-CORT in a fast-acting time-course that parallels the effects of ketamine (Scheil et al., 2024). This highlights the importance of evaluating the molecular mechanisms underlying the central and behavioral effects of a single peripheral injection of recombinant reelin, which will be a focus of future research stemming from this study.

All in all, we showed that the intravenous injection of the reelin central fragment ameliorates PPD by improving despair-like behavior, as evidenced by a reduction in immobility time in the FST and restored some brain neurochemical alterations associated with depression. While PPD encompasses a range of symptoms beyond despair, the FST is a widely accepted method for evaluating antidepressant efficacy because all approved antidepressants decrease immobility time without increasing general locomotor activity. This makes the FST a reliable marker of predictive validity for antidepressant effects. Reelin treatment’s efficacy in this model suggests that it may help restore normal brain function disrupted by chronic stress during the post-partum period. However, to confirm that reelin treatment is genuinely effective for PPD, it is necessary to conduct additional tests to measure other behavioral and neurochemical alterations associated with depressive phenotypes in humans. Specifically, future research should aim to assess other behavioral changes in treated animals to ensure a comprehensive understanding of reelin’s effects. Investigate neurochemical markers typical of depression in human subjects, such as neurotransmitter levels and receptor expressions. Evaluate the long-term effects of reelin treatment on both behavior and brain chemistry to ensure sustained benefits. These steps will provide a more detailed understanding of how reelin treatment can alleviate PPD and help validate its potential as a therapeutic option.

## 5 Conclusion

This study provides additional evidence supporting the involvement of oxytocin-positive cells in the PVN, reelin-positive cells and neuronal maturation in the hippocampal SGZ in the regulation of HPA axis responses to stress during the post-partum period. Additionally, we present data indicating that peripheral reelin administration restores behavioral and neurochemical alterations induced by chronic corticosterone exposure, showing novel antidepressant-like effects of reelin in a preclinical model for post-partum depression. This research holds significance, especially since various stress-related psychiatric conditions are associated with disturbances in the functioning of both the HPA axis and the oxytocin system. Further investigations must be conducted following these findings to gain a comprehensive understanding of the role of reelin in modulating post-partum behavior.

## Data Availability

The raw data supporting the conclusions of this article will be made available by the authors, without undue reservation.

## Glossary

ANOVA: Analysis of Variance
BBB: Blood–Brain Barrier
BSA: Bovine Serum Albumin
CI: Confidence Interval
CORT: Corticosterone
C/R: CORT-Reelin
C/V: CORT-Vehicle
DCX: Doublecortin
DSM-5: Diagnostic and Statistical Manual of Mental Disorders, Fifth Edition
EPM: Elevated Plus Maze
FST: Forced Swim Test
HPA: Hypothalamic-Pituitary-Adrenal Axis
η²_p_: Partial Eta-Squared
IQR: Interquartile Range
OXT: Oxytocin
PBS: Phosphate Buffer Saline
PD: Post-Partum Day
PFA: Paraformaldehyde
PVN: Paraventricular nucleus
SGZ: Subgranular Zone
S/R: Saline-Reelin
S/V: Saline-Vehicle
TBS: Tris-Buffered Saline
VL: Ventrolateral
VD: Ventrodorsal
