# Supplementary material for: A single intravenous reelin injection restores corticosterone-induced neurochemical and behavioral alterations in dams during the post-partum period
Source: Front Mol Neurosci. 2024 Aug 20;17:1442332. doi: 10.3389/fnmol.2024.1442332 (PMC11369980; doi:10.3389/fnmol.2024.1442332)
Supplement: Supplementary file 1 [file Table_1.docx]

**Supplementary figures and tables:**

**Supplementary Figure 1.** **Estrous cycle and peripheral estradiol levels.** A) Estradiol levels in serum are the same between treatment groups, B) regardless of the estrous cycle stage of the rat. Mean ± 95%CI.

**Supplementary Table 1. Treatment effects on maternal behaviour.** Statistical significance *p<0.05, **p<0.01, ***p<0.001

| **Between Subjects Effects** | **Statistics** | **Treatment 1 (CORT)** | **Treatment 2 (litter size)** | **Treatment 1 & treatment 2** |
| --- | --- | --- | --- | --- |
| Time nursing | F(1,24) | 3.741 | 0.563 | 4.081 |
|  | P-value | 0.066 | 0.461 | 0.056 |
|  | η²_p_ | 0.145 | 0.025 | 0.156 |
| Time offnest | F(1,24) | 8.689 | 0.525 | 0.105 |
|  | P-value | 0.007** | 0.476 | 0.749 |
|  | η²_p_ | 0.283 | 0.023 | 0.005 |
| Time self-grooming | F(1,24) | 0,068 | 5,020 | 0,685 |
|  | P-value | 0,796 | 0,035* | 0,417 |
|  | η²_p_ | 0,003 | 0,186 | 0,030 |
| Time licking and grooming pups | F(1,24) | 0,045 | 1,128 | 0,261 |
|  | P-value | 0,833 | 0,300 | 0,615 |
|  | η²_p_ | 0,002 | 0,049 | 0,012 |

**Supplementary Table 2:** Statistical information (2-way ANOVA) for FST and EPM behaviour.

| **Between Subjects Effects** | **Statistics** | **Treatment 1 (CORT)** | **Treatment 2 (Reelin)** | **CORT x Reelin** |
| --- | --- | --- | --- | --- |
| Immobility | F(1,24) | 17.351 | 22.520 | 1.183 |
|  | P-value | 0.0001*** | 0.0001*** | 0.288 |
|  | η²_p_ | 0.420 | 0.484 | 0.047 |
| Climbing | F(1,24) | 7.108 | 7.95 | 0.010 |
|  | P-value | 0.014 * | 0.009 ** | 0.922 |
|  | η²_p_ | 0.249 | 0.228 | 0.000 |
| Swimming | F(1,24) | 0.118 | 0.026 | 0.569 |
|  | P-value | 0.734 | 0.872 | 0.458 |
|  | η²_p_ | 0.005 | 0.001 | 0.023 |
| Latency to immobility | F(1,24) | 3.455 | 0.388 | 0.001 |
|  | P-value | 0.075 | 0.539 | 0.978 |
|  | η²_p_ | 0.126 | 0.016 | 0.000 |
| Time open arms | F(1,24) | 0.819 | 0.000 | 0.128 |
|  | P-value | 0.375 | 0.983 | 0.723 |
|  | η²_p_ | 0.033 | 0.000 | 0.005 |
| Distance travelled | F(1,24) | 0.008 | 3.347 | 3.044 |
|  | P-value | 0.929 | 0.080 | 0.094 |
|  | η²_p_ | 0.000 | 0.127 | 0.117 |

**Supplementary Table 3.** Statistical information (2-way ANOVA) for reelin-positive cells in the PVN and hippocampus SGZ.

| **Between Subjects Effects** | **Statistics** | **Treatment 1 (CORT)** | **Treatment 2 (Reelin)** | **CORT x Reelin** |
| --- | --- | --- | --- | --- |
| PVN (VD+VL) | F(1,24) | 2.143 | 2.193 | 2.813 |
|  | P-value | 0.152 | 0.152 | 0.106 |
|  | η²_p_ | 0.084 | 0.084 | 0.105 |
| PVN-VD | F(1,24) | 2.704 | 4.590 | 3.302 |
|  | P-value | 0.113 | 0.043* | 0.082 |
|  | η²_p_ | 0.082 | 0.101 | 0.121 |
| PVN-VL | F(1,24) | 2.465 | 3.207 | 1.070 |
|  | P-value | 0.130 | 0.086 | 0.311 |
|  | η²_p_ | 0.093 | 0.118 | 0.043 |
| dHP | F(1,24) | 14.280 | 1.683 | 0.330 |
|  | P-value | 0.001** | 0.207 | 0.571 |
|  | η²_p_ | 0.373 | 0.066 | 0.014 |
| iHP | F(1,24) | 15.592 | 0.606 | 6.912 |
|  | P-value | 0.001** | 0.444 | 0.015* |
|  | η²_p_ | 0.394 | 0.025 | 0.224 |
| vHP | F(1,24) | 8.571 | 0.310 | 1.262 |
|  | P-value | 0.007** | 0.583 | 0.272 |
|  | η²_p_ | 0.263 | 0.013 | 0.050 |

**Supplementary Table 4.** Statistical information (2-way ANOVA) for oxytocin-positive cells in the PVN.

| **Between Subjects Effects** | **Statistics** | **Treatment 1 (CORT)** | **Treatment 2 (Reelin)** | **CORT x Reelin** |
| --- | --- | --- | --- | --- |
| PVN (VD+VL) | F(1,24) | 3.997 | 0.446 | 3.498 |
|  | P-value | 0.057 | 0.511 | 0.074 |
|  | η²_p_ | 0.143 | 0.018 | 0.127 |
| PVN-VD | F(1,24) | 0.438 | 0.105 | 1.707 |
|  | P-value | 0.514 | 0.748 | 0.204 |
|  | η²_p_ | 0.018 | 0.004 | 0.066 |
| PVN-VL | F(1,24) | 12.215 | 44.098 | 3.861 |
|  | P-value | 0.002** | 0.0001**** | 0.061 |
|  | η²_p_ | 0.337 | 0.648 | 0.139 |

**Supplementary Table 5.** Statistical information (2-way ANOVA) for DCX-positive cells in the dHP, including categories and developmental stage.

| **Between Subjects Effects** | **Statistics** | **Treatment 1 (CORT)** | **Treatment 2 (Reelin)** | **CORT x Reelin** |
| --- | --- | --- | --- | --- |
| dHP | F(1,24) | 6.023 | 2.113 | 11.082 |
|  | P-value | 0.022* | 0.159 | 0.003** |
|  | η²_p_ | 0.201 | 0.081 | 0.316 |
| % Cat 1 | F(1,24) | 14.777 | 0.071 | 1.577 |
|  | P-value | 0.001** | 0.792 | 0.221 |
|  | η²_p_ | 0.381 | 0.003 | 0.062 |
| % Cat 2 | F(1,24) | 26.211 | 0.235 | 0.479 |
|  | P-value | 0.0001**** | 0.633 | 0.496 |
|  | η²_p_ | 0.522 | 0.010 | 0.020 |
| % Cat 3 | F(1,24) | 0.866 | 0.096 | 1.039 |
|  | P-value | 0.361 | 0.759 | 0.318 |
|  | η²_p_ | 0.035 | 0.004 | 0.042 |
| % Cat 4 | F(1,24) | 5.150 | 1.025 | 0.206 |
|  | P-value | 0.033* | 0.321 | 0.654 |
|  | η²_p_ | 0.177 | 0.041 | 0.009 |
| % Cat 5 | F(1,24) | 25.663 | 0.856 | 0.701 |
|  | P-value | 0.00001**** | 0.364 | 0.411 |
|  | η²_p_ | 0.517 | 0.034 | 0.028 |
| % Cat 6 | F(1,24) | 13.093 | 2.095 | 3.129 |
|  | P-value | 0.001** | 0.161 | 0.090 |
|  | η²_p_ | 0.353 | 0.080 | 0.115 |
| % proliferative | F(1,24) | 28.004 | 0.199 | 1.269 |
|  | P-value | 0.0001**** | 0.659 | 0.271 |
|  | η²_p_ | 0.538 | 0.008 | 0.050 |
| % intermediate | F(1,24) | 1.781 | 1.192 | 0.092 |
|  | P-value | 0.195 | 0.286 | 0.764 |
|  | η²_p_ | 0.069 | 0.047 | 0.004 |
| % postmitotic | F(1,24) | 30.946 | 2.392 | 2.923 |
|  | P-value | 0.0001**** | 0.135 | 0.100 |
|  | η²_p_ | 0.563 | 0.091 | 0.109 |

**Supplementary Table 6.** Statistical information (2-way ANOVA) for DCX-positive cells in the iHP, including categories and developmental stage.

| **Between Subjects Effects** | **Statistics** | **Treatment 1 (CORT)** | **Treatment 2 (Reelin)** | **Treatment 1 & treatment 2** |
| --- | --- | --- | --- | --- |
| DCX+ cells iHP | F(1,24) | 0.530 | 8.308 | 6.538 |
|  | P-value | 0.474 | 0.008** | 0.017* |
|  | η²_p_ | 0.022 | 0.257 | 0.214 |
| % Cat 1 | F(1,24) | 17.207 | 0.628 | 14.253 |
|  | P-value | 0.0001**** | 0.436 | 0.001** |
|  | η²_p_ | 0.418 | 0.025 | 0.373 |
| % Cat 2 | F(1,24) | 1.074 | 4.133 | 4.877 |
|  | P-value | 0.310 | 0.054 | 0.037* |
|  | η²_p_ | 0.043 | 0.146 | 0.169 |
| % Cat 3 | F(1,24) | 0.149 | 2.211 | 0.367 |
|  | P-value | 0.703 | 0.150 | 0.550 |
|  | η²_p_ | 0.006 | 0.084 | 0.015 |
| % Cat 4 | F(1,24) | 0.306 | 4.895 | 0.000 |
|  | P-value | 0.585 | 0.037 | 1.000 |
|  | η²_p_ | 0.013 | 0.169 | 0.000 |
| % Cat 5 | F(1,24) | 12.039 | 0.107 | 25.410 |
|  | P-value | 0.002** | 0.746 | 0.0001**** |
|  | η²_p_ | 0.334 | 0.004 | 0.514 |
| % Cat 6 | F(1,24) | 11.622 | 9.414 | 29.753 |
|  | P-value | 0.002** | 0.005* | 0.0001**** |
|  | η²_p_ | 0.326 | 0.282 | 0.554 |
| % proliferative | F(1,24) | 13.430 | 4.116 | 18.094 |
|  | P-value | 0.001** | 0.054 | 0.0001**** |
|  | η²_p_ | 0.359 | 0.146 | 0.430 |
| % intermediate | F(1,24) | 0.431 | 0.526 | 0.144 |
|  | P-value | 0.518 | 0.475 | 0.707 |
|  | η²_p_ | 0.018 | 0.021 | 0.006 |
| % postmitotic | F(1,24) | 17.195 | 3.342 | 39.525 |
|  | P-value | 0.0001**** | 0.080 | 0.0001**** |
|  | η²_p_ | 0.417 | 0.122 | 0.109 |

**Supplementary Table 7.** Statistical information (2-way ANOVA) for DCX-positive cells in the vHP, including categories and developmental stage.

| **Between Subjects Effects** | **Statistics** | **Treatment 1 (CORT)** | **Treatment 2 (Reelin)** | **Treatment 1 & treatment 2** |
| --- | --- | --- | --- | --- |
| DCX+ cells iHP | F(1,24) | 7.565 | 11.832 | 5.094 |
|  | P-value | 0.011* | 0.002** | 0.034* |
|  | η²_p_ | 0.248 | 0.340 | 0.181 |
| % Cat 1 | F(1,24) | 27.065 | 4.678 | 15.338 |
|  | P-value | 0.0001**** | 0.041 | 0.001 |
|  | η²p | 0.530 | 0.163 | 0.390 |
| % Cat 2 | F(1,24) | 45.493 | 2.483 | 15.771 |
|  | P-value | 0.0001**** | 0.128 | 0.001 |
|  | η²_p_ | 0.655 | 0.094 | 0.397 |
| % Cat 3 | F(1,24) | 1.939 | 1.697 | 4.844 |
|  | P-value | 0.177 | 0.205 | 0.038* |
|  | η²_p_ | 0.075 | 0.066 | 0.168 |
| % Cat 4 | F(1,24) | 0.244 | 2.193 | 1.911 |
|  | P-value | 0.626 | 0.152 | 0.180 |
|  | η²_p_ | 0.010 | 0.084 | 0.074 |
| % Cat 5 | F(1,24) | 21.609 | 0.368 | 12.368 |
|  | P-value | 0.0001**** | 0.550 | 0.002** |
|  | η²_p_ | 0.474 | 0.015 | 0.340 |
| % Cat 6 | F(1,24) | 45.432 | 9.240 | 17.886 |
|  | P-value | 0.0001**** | 0.006** | 0.0001**** |
|  | η²_p_ | 0.654 | 0.278 | 0.427 |
| % proliferative | F(1,24) | 54.698 | 5.473 | 24.063 |
|  | P-value | 0.0001**** | 0.028* | 0.0001**** |
|  | η²_p_ | 0.695 | 0.186 | 0.501 |
| % intermediate | F(1,24) | 0.635 | 0.001 | 0.635 |
|  | P-value | 0.433 | 0.970 | 0.433 |
|  | η²_p_ | 0.026 | 0.0001 | 0.026 |
| % postmitotic | F(1,24) | 35.281 | 2.788 | 17.084 |
|  | P-value | 0.0001**** | 0.108 | 0.0001**** |
|  | η²_p_ | 0.595 | 0.104 | 0.416 |
